# Looks at what isn't there: eye movements on a blank screen when processing negation in a first and a second language

**DOI:** 10.3389/fnhum.2024.1457038

**Published:** 2024-10-10

**Authors:** Norbert Vanek, Ana Matić Škorić, Sara Košutar, Štěpán Matějka, Kate Stone

**Affiliations:** ^1^School of Cultures, Languages and Linguistics, The University of Auckland, Auckland, New Zealand; ^2^Department of Speech and Language Pathology, University of Zagreb, Zagreb, Croatia; ^3^Department of Language and Culture, UiT The Arctic University of Norway, Tromsø, Norway; ^4^Institute of Czech Language and Theory of Communication, Charles University, Prague, Czechia; ^5^Department of Linguistics, University of Potsdam, Potsdam, Germany

**Keywords:** negation processing, anticipatory eye movements, blank screen paradigm, Croatian, English

## Abstract

Is negation more difficult to process than affirmation? If it is, does processing negation in a second language (L2) compound the difficulty compared to the first language (L1)? This article addresses the issues of difficulties in processing different types of negation in the L1 and L2 by looking at the differences in the ways in which comprehenders anticipate upcoming visual information during sentence processing. Using a blank-screen paradigm, we recorded eye fixations of Croatian native speakers and Croatian learners of English while they were anticipating matching or mismatching pictures to sentences with various types of negation in L1 (Croatian) and L2 (English). Using a between-group design, we manipulated sentence polarity (affirmative vs. negative), negation type (sentential vs. negative quantifier) within both L1 Croatian and L2 English so that we could observe potential anticipation effects varying as a function of the two predictors. In line with previous studies, affirmation in the L1 was easier to process than negation, and participants were able to anticipate sentence-picture matches in both the L1 and the L2 group. In contrast with our prediction, anticipatory looks did not significantly vary across negation types in Croatian based on the number of structural cues. In L2 English, learners exhibited prediction ability across negation types. These findings go against the view that comprehension in L2 comes with a reduced ability to generate expectations, and they highlight the robustness of mental simulations in both L1 and L2 negation processing.

## 1 Introduction

Research on processing negation in a native language, especially negation with no or little context, has reached a fairly well-established consensus that the processing of negative statements is cognitively more demanding than the processing of affirmative ones (e.g., Carpenter and Just, 1975; Coso and Bogunović, 2016; Kaup, 2001; Kaup et al., 2006, 2007; MacDonald and Just, 1989; Orenes et al., 2022). Greater cognitive effort needed to process negation than affirmation comes from a range of measures and paradigms, including picture-sentence verification (Coso and Bogunović, 2016), recognizing probes embedded in isolated sentences (MacDonald and Just, 1989) or in narrative texts (Kaup, 2001), self-paced reading followed by naming picture matches (Kaup et al., 2006), or monitoring eye fixations on pictures while hearing negative or affirmative sentences (Orenes et al., 2014). Nevertheless, there are certain aspects that lack consensus, including whether individuals mentally construct a direct representation of the negated state (e.g., a whole onion when hearing ‘*nobody chopped the onion*') or if negation processing necessitates an extra step as a detour through mentally representing the corresponding positive state first (a chopped onion) as suggested by Kaup et al. (2006, 2007). This theory posits that these two steps are an essential sequence to follow for negation to be understood. Numerous studies found support for this indirect way in the form of increased processing demands associated with the negated state of affairs compared to the positive alternative (e.g., Dudschig and Kaup, 2018; Hasson and Glucksberg, 2006; Kaup et al., 2007; Tian et al., 2016). Indirect processing of negation via the positive alternative aligns more broadly with the embodied cognition theory, which assumes that mental representations originate from direct sensorimotor experience with the world (Barsalou, 1999; Varela et al., 1991). Under the indirect view, when listeners process negation as in “Nobody broke the coconut”, they would first mentally simulate the positive alternative (a broken coconut), and then proceed to the simulation of the negated state (a whole coconut). In contrast, the alternative view is that automatic, direct processing of the negated states of affairs happens without the need to initially represent the alternative positive state of affairs. This view also enjoys substantial empirical support (e.g., Mayo et al., 2004; Orenes et al., 2014; Tian et al., 2010). Direct processing of negation aligns with the view of reliance of mental representations on abstract, symbolic mental computations (Pylyshyn, 1986; Firestone and Scholl, 2016) independent from the physical body's interactions with the environment.

A somewhat less explored area of research pertains to crosslinguistic differences in the processing of negation due to variation in structural encoding (Dudschig et al., 2021; Zhang and Vanek, 2021). One negation type that has attracted considerable research interest is *negative concord* (Coso and Bogunović, 2016; Déprez et al., 2015; Giannakidou, 2000; Maldonado and Culbertson, 2021). In negative concord, a sentence has two or more negative elements, but these elements yield only one semantic negation (Giannakidou, 2020; Zeijlstra, 2007), as in the Croatian example *Nitko nije vidio dječaka* “Nobody saw the boy”. Negative concord boasts great crosslinguistic variation. In some languages, like in Croatian, this type of negation is obligatory, ^*^Nitko je vidio dječaka “Nobody saw the boy”, while in others, negative concord is ungrammatical, like in standard English ^*^Nobody didn't see the boy (Robinson and Thoms, 2021). Moreover, languages with negative concord come in different guises. Following Giannakidou (2000), negative concord languages can be placed on a continuum depending on how strongly the negative marker is required to accompany the negative quantifier. In *strict negative concord* languages, such as most Slavic languages or Greek, a negative quantifier requires the co-presence of a negative marker, regardless of the position in a sentence (e.g., in Croatian, *Nitko nije zvao*, lit. “Nobody not called”). However, in *non-strict negative concord* languages, such as Spanish or Italian, the negative quantifier can occur without the negative marker in a preverbal position (e.g., in Italian, *(Non) ha chiamato nessuno* lit. “(Not) called nobody”). And as the third group, languages that do not readily allow negative concord are called *double negation languages* (Zeijlstra, 2007). In double (or multiple) negation, a sentence contains two (or more) negative elements that cancel each other out, resulting in an affirmation, such as in the English *I don't like no sci-fi* (meaning “I like some sci-fi”).

This study targets processing in two languages, one with negative concord (Croatian), and the other without it (English). Croatian is a strict negative concord language (Zovko Dinković, 2013, 2021). A common way to form a negative sentence is by negating a universal quantifier, for example, *svatko* “everybody” in *Svatko je vidio dječaka*. “Everybody saw the boy” is negated into the negative quantifier *nitko* “nobody” in *Nitko nije vidio dječaka*. lit. “Nobody didn't see the boy”. The timecourse of processing negative concord still remains largely unknown. The nature of the timecourse is important and informative when claims are formulated about incremental processing. Negative concord is an unique type of negation from a processing perspective, particularly when the first language requires it, but the second language rules it out, at least in its standard variety. Although negative concord has traditionally been regarded as grammatically incorrect in standard English, recent research suggests that its acceptability and comprehension can vary depending on context (Blanchette and Lukyanenko, 2019). For instance, contextual cues can bias the reading of *She didn't answer nothing in that interview* not only as double negation (“she answered something”) but also as negative concord (“she answered nothing”). In this study, we integrate a layer of negation processing in standard English as a second language with the aim to explore processing patterns when the L1 and L2 structures differ.

## 2 Empirical context: negation processing in L2

Research on processing negation in a second language is teeming with mixed results. Some studies report additional processing costs incurred by L2 learners compared to native speakers when they process negation (e.g., Hasegawa et al., 2002; Manning et al., 2018), while others find little or no added difficulty for negation processing in a second language (Coso and Bogunović, 2019; Zhang and Vanek, 2021; Zhang et al., 2022). We first survey representative neuroimaging research with L2 speakers, which points to L1-L2 differences in negation processing. For instance, Hasegawa et al. (2002) used fMRI to compare cortical activity in native Japanese speakers during the auditory processing of sentences in L1 Japanese and L2 English. The analyses showed that the L2 required more cognitive effort and computation from the shared network of cortical regions than the L1, and that negative sentences in English elicited greater activation, indicating that the structural difficulty of negation has a larger impact on cortical activation if it occurs in the context of the second language. One limitation that the present study addressed is the poor temporal resolution of fMRI data to uncover potential differences in the timecourse of L1 and L2 language processing. An L2 disadvantage was also reported by Manning et al. (2018), who examined how L1 and L2 English speakers process negation, using a more time-sensitive EEG method. The participants were French learners of English and simultaneous French–English bilinguals, who were asked to read true/false positive/negative sentences (e.g., *The jury found him innocent/guilty because the fire was recognized as intentional/not intentional in court*) while ERPs were recorded. A greater N400 was observed in L2 for true negatives (…*innocent* …*not intentional*…) than for true positives (…*guilty …intentional*…), but no such difference emerged in simultaneous bilinguals. The authors reasoned that the discrepancy reflected additional processing costs in the context of a second language. One limitation in Manning et al. (2018) is its focus on just a single type of negation. Inclusion of different negation types in the present study addresses this limitation.

Some studies reported little or no differences in L1 and L2 processing. We look at a portion of representative research that used behavioral methods. For instance, Coso and Bogunović (2019) found no increased difficulty in reaction time and accuracy measures in Croatian learners of English. They used a sentence-picture verification task [e.g., *Hearts are (not) above an arrow*] and compared reaction times for affirmation and negation in L1 and L2. The study tested different negation types in both languages, namely sentential negation and constituent negation, Croatian negative concord, and English sentences with negated subject, all manipulated within participants. Such an elegant design enabled direct comparisons between the processing implications of different structural cues specific for English and Croatian. Among the key findings were that negative concord and sentences with a negated subject had similar reaction times (M = 2,180.12, SD = 162.32) as sentential negation (M = 2,077.99, SD = 139.74), but significantly higher accuracy (negative concord: M = 92.59%, SE = 1.60; sentential negation 79.40%, SD = 1.85). The authors interpreted the accuracy advantage found for negative concord as support for the idea that strong cues, such as in this case a negative universal quantifier in addition to a negated verb in Croatian negative concord, facilitate language processing (Coso and Bogunović, 2019, p. 32). Nevertheless, one may argue that the reason for the difference found in accuracy should not necessarily be assigned to easier or more accurate processing of negative concord, but to the relative vagueness of the sentences used in the task design, which are not very common in everyday communication amongst Croatian speakers. Also, while reaction time (RT) measures for different negation types are in a good position to capture cue-driven processing difficulty, RTs are not well positioned to arbitrate between the one-step vs. two-step approach still resonant in the negation processing literature. The present study adopts a visual world eye-tracking design using the blank screen method (Altmann, 2004) to track whether listeners process negation directly or through an initial detour via the corresponding positive state of affairs. An additional theory-building potential of the eye-tracking approach is to show the extent to which second language learners can generate expectations depending on language-specific cues.

The blank-screen method builds on the findings from the visual world paradigm (Tanenhaus et al., 1995) that individuals launch eye fixations to a relevant picture in tight synchrony with the timing of the corresponding spoken expression. For instance, when individuals see a picture showing a woman, newspaper, a cake, and a man, as soon as they hear ‘*The woman will read the …*' their eyes tend to fixate on the newspaper even before the target object gets mentioned, in line with the selection restrictions of the verb (Altmann and Kamide, 1999). Such fixations are known as *anticipatory eye movements*. They signal that the processing system can make quick language-modulated predictions about the upcoming expression based on concurrent visual input. However, anticipatory eye movements do not depend on the physical presence of visual input, they were observed even when the previously introduced visual scene got removed (Altmann, 2004; Richardson and Spivey, 2000). To illustrate, Altmann (2004) showed English native speakers visual scenes before playing them the corresponding sentences, and found that linguistic input triggered anticipatory fixations even when the screen was blank. Anticipatory fixations on a blank screen largely mirrored the eye movement patterns observed when linguistic and visual information were presented simultaneously. Fixations launched on the blank screen, before hearing the target referring expression (e.g., newspaper), signal that individuals mentally simulate visual scenes and launch anticipatory looks toward the target item irrespective of whether the scenes are shown or absent.

## 3 Theoretical grounding

One prominent account of potential differences in L1 and L2 processing, which directly addresses the question of whether the ability to compute expectations differs based on native vs. non-native language context, is the *Reduced Ability to Generate Expectations* (RAGE) hypothesis (Grüter et al., 2014). It builds on the assumption that, unlike native speakers, L2 learners have generally suboptimal abilities to make use of cues from the input stream to generate predictions about what is coming next. As follows from RAGE, a reduced (or no) ability to predict as native speakers do could be attributed to the differences in how linguistic information is processed. While for native listeners prediction (anticipatory processing) is key to successfully comprehend input as it enables fast message decoding and early response planning for a smooth flow of conversations (Pickering and Garrod, 2007), non-native listeners may be more likely to process a word only after it has appeared (integratory processing).

Evidence for RAGE was found in behavioral L2 studies, some of which used eye-tracking. For instance, Hopp (2013) tested sentence processing in native German speakers and advanced English learners of German. Visual world eye-tracking was used with sentences including articles marked for gender, serving as the predictive cue for the upcoming noun. Learners did not reach the extent to which native speakers used the structural cue to launch anticipatory looks toward the target, which led to the conclusion that L2 processing is characterized by a reduced predictive ability. Another example comes from Van Bergen and Flecken (2017), who measured anticipatory eye movements to objects while French and German learners of Dutch and Dutch native speakers were listening to Dutch sentences with placement verbs (put.STAND vs. put.LIE). German typically specifies position in placement verbs while French usually does not. The results showed that German learners and Dutch native speakers could anticipate the object that matched the position encoded in the verb, but French learners did not exhibit any prediction effects even though they too understood the verbs. This finding was interpreted as evidence that L2 learners have difficulty using lexical cues for prediction, but only when the key linguistic feature differs across the listener's two languages. In terms of mental simulation in L2 processing, these findings from predictive looks point to an overall reduced effect compared to L1 processing.

Evidence against RAGE was found in behavioral as well as neurophysiological studies. Comparable anticipation ability in native speakers and L2 learners was observed where the linguistic features of the source and the target language overlap (e.g., Dussias et al., 2013; Foucart et al., 2014). To illustrate, Foucart et al. (2014) examined lexical prediction ability through monitoring brain activity (event-related potentials) in French learners of Spanish, Spanish-Catalan bilinguals and Spanish native speakers. The key manipulation was in sentence endings, in which the critical noun was either expected or not. Nouns (expected vs. unexpected) varied in gender so that potential anticipation effects could emerge on the article. Anticipation effects (N400 modulations) were found across the groups, suggesting that second language listeners can use cues to predict upcoming input, at least when the L1 and L2 are similar.

The literature on predictive processing in a second language, measured through anticipatory looks in particular, boasts an impressive array of studies. Areas of inquiry span from semantic prediction of the upcoming noun after hearing the verb (e.g., Chambers and Cooke, 2009; Dijkgraaf et al., 2019) to prediction of the upcoming noun using morpho-syntactic cues to signal agreement relationships (e.g., Dussias et al., 2013; Lago et al., 2023). However, prediction during negation processing, both in the L1 and L2, has so far remained unexplored even though this combination could potentially be useful for theory building. Negation in L2 offers an informative new kind of test of the RAGE hypothesis because negation is more universal (compared to e.g., grammatical gender) but encoded differently across languages. This property allows us to test whether anticipation effects in L2 negation processing surface at all, and if they do, whether they are present only when the L1 and L2 structurally overlap. Particularly important is the scenario when the L1 with two negative cues may or may not influence processing in L2 with just a single cue. Regarding L2 prediction ability, the aim of this study is to probe into the nature of anticipatory and integratory processing of negation by examining listeners' ability to launch eye fixations toward the correct target before and after it has turned up. Testing listeners in their native and non-native language is advantageous for establishing whether, and if so, then how fast, they can use language-specific cues predictively.

Another prominent account of potential differences in L1 and L2 processing, compatible with RAGE, is the *Competition Model* (MacWhinney, 1987). This model foregrounds the role of language-specific cues in processing and learning a language. It assumes that greater strength of linguistic cues will contribute to shorter reaction times and greater processing accuracy. In its extended version, the Unified Competition Model (UCM) (MacWhinney, 2005, 2008, 2012), views the mechanism of L2 processing and learning as L2 forms entering mental maps that are already strongly committed to L1 patterns, and therefore they align with analogous L1 forms. If the structures in L1 and L2 are aligned, cue validity is enhanced. But if the two structures do not match, cue validity decreases. Thus, if there is a significant structural difference between the two languages, processing the L2 will be more difficult. Some interference is predicted even for highly proficient L2 speakers (MacWhinney, 2002) as the two grammars interact during cognitive processing. Following the logic of increased cue validity based on L1-L2 structural alignment, cue strength differences can be expected when L1 Croatian learners of L2 English process various types of negation. One can expect negative concord with two negative items in Croatian and the equivalent single item negation in English to make negation processing more difficult and less accurate for Croatian learners of L2 English compared to sentential negation with a single negative item in both languages. Alternatively, learners may develop separate L2-based processing routines not characterized by L1–L2 structural alignment.

## 4 The present study

This study fills two research gaps, one theoretical and one methodological. From a theoretical point of view, we were interested in examining whether L2 learners generate expectations that are sensitive to the structural cues of each of their languages. This layer of analysis is particularly important for understanding the computations involved in sentence processing, especially given the variation in which negation is encoded in Croatian and English. Methodologically, we extended previous experimental procedures in the area (e.g., reaction time measures indicative of incremental processing but not of mental simulations) to anticipatory eye movements using the blank screen paradigm (Altmann, 2004), thus tapping into the various stages of processing as they unfold during language-modulated mental simulations. While the blank screen paradigm was found suitable in earlier research which examined negation processing crosslinguistically [Croatian L1 vs. English L1 in Vanek et al. (2024)], to the best of the authors' knowledge the current study is the first extension of this paradigm to second language processing research.

The structural cues of our primary interest were negative concord in Croatian and its nearest equivalent, negative quantifier negation in English. If Croatian second language learners of English compute predictions depending on the currently used language, one would expect language-specific variation in processing patterns. Our main motivation was to find out whether Croatian learners of English process corresponding negation structures across their two languages (negative concord in Croatian and negative quantifier in English) differently or not. The rationale for potentially different processing patterns across negations was that negative concord in L1 Croatian provides a double cue that might facilitate the process of generating predictions about the factual state of affairs more strongly than sentential negation, where Croatian only provides a single cue. A possible alternative for L1 Croatian is that the two negative items in negative concord may be an instance of redundancy (e.g., Zovko Dinković, 2021), in which case negative concord would not be expected to boost predictions compared to sentential negation.

Two research questions were tested, one focussing on within-language differences for various negation types, and the other on the processing of comparable negation structures across the speakers' L1 and L2. The first research question asked if there are any marked differences in the processing patterns that characterize various types of negations within L1 Croatian and within L2 English. Within Croatian, our hypothesis following the Competition Model was that generation of expectations about the upcoming input will be faster in negative concord sentences than in sentential negation. This hypothesis is grounded more firmly in the *cue salience account* (Ellis and Sagarra, 2010; Sagarra and Ellis, 2013; Ellis, 2017), which posits that differences in perceptual salience of linguistic forms lead to variation in their processing. Under this account, more lexical/morpho-phonetic substance in the functor (Ellis, 2017, p. 80), such as two lexical items in the encoding of negative concord compared to just one lexical item in sentential negation, can predict easier processing. Following the RAGE hypothesis (Grüter et al., 2014), no such differences were predicted for English as L2. The second research question asked if there are parallels in the processing of negation between Croatian (L1) and English (L2), namely if the differences between two types of negations per language are comparable across the participant's native and non-native languages. Across languages, following the cue salience account, we expected greater differences in the processing of different types of negation structures in L1 Croatian (negative concord vs. sentential negation, i.e., double vs. single cue respectively) compared to L2 English (negative quantifier vs. sentential negation, i.e., single vs. single cue). For the purpose of apt and consistent label use, in this study, factual denotes the negated state of affairs (e.g., intact balloon for *Nobody pierced the balloon*) and illusory refers to the positive alternative (pierced balloon).

## 5 Experiment 1: processing negation in Croatian L1

### 5.1 Participants

The sample consisted of 32 native Croatian speakers (*MAGE* = 22.7; *SD*AGE = 1.5; 28 females). All participants were students recruited at a university in Croatia. To determine sample size adequacy, G^*^Power (Erdfelder et al., 1996) was used to check the sample size needed to test the main prediction that anticipatory looks toward the factual would significantly exceed anticipatory looks toward the illusory, both in sentential negation and in double negation. For a power of 0.8 with a medium effect size estimate of *d* = 0.5 and a significance threshold of α = 0.05 for two dependent means, the sample size suggestion was *N* = 34. During the tests, the number of valid recordings slightly attrited to 32. All participants reported Croatian as their native and dominant language, normal or corrected-to-normal vision, and no history of neurological and/or language impairments. They had learned English since elementary school and throughout high school (total of ~12 years). Their English proficiency was assessed using the Oxford Placement Test 1, level B2 (upper-intermediate level) (Allan, 1985). The average score of the group was 43 (out of 50), with a minimum of 36 points and a maximum of 47 points, indicating that they all had at least a B2 level. In addition, all students reported daily exposure to English through formal education and social media. Based on the test results, they were treated as a group of advanced users of English as a second language. The study was approved by the Ethics Committees of the University of Auckland. Before the experiment, all participants gave written informed consent to take part in the study. Participation was compensated with a gift voucher.

### 5.2 Materials

**Figure 1:**
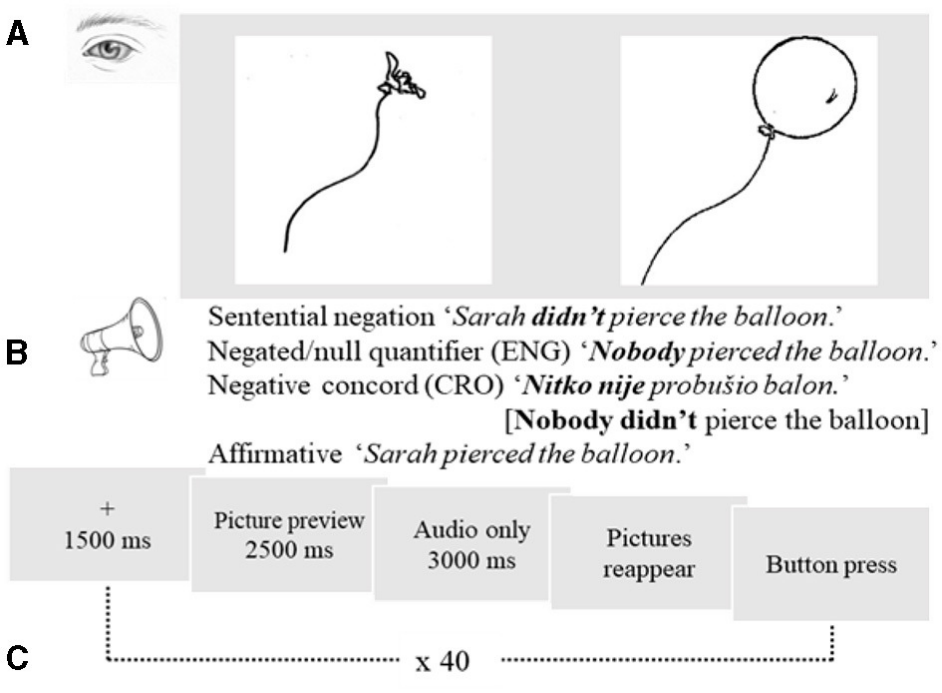
Experiment design. **(A)** A picture pair showing an illusory vs. factual alternate. **(B)** Audio-recorded linguistic input in Croatian varying between sentential negation, negative concord, and an affirmative; and in English varying between sentential negation, negative quantifier negation, and an affirmative. **(C)** A trial sequence including a fixation cross, picture preview, blank screen with audio input, followed by pictures reappearing in their original positions and shown until button press.

The stimuli consisted of sets of audio recordings and corresponding picture pairs (Figure 1). The audios were pre-recorded sentences, all of the same length (3,000 ms), read out by a native speaker of Croatian. The audios served as linguistic stimuli including three different structure types. These were sentential negation, negative concord, and affirmative controls, all presented in Croatian. A total of twenty pairs of pictures were combined with the three different sentence types. Examples of the sentence types in both languages are (a) sentential negation in Croatian (*Sara nije probušila balon*) and English (Sarah didn't pierce the balloon); (b) negative concord in Croatian (*Nitko nije probušio balon;* ^*^Nobody didn't pierce the balloon') and the corresponding negative quantifier negation in English (*Nobody pierced the balloon*); and (c) affirmative sentences in Croatian (*Sara je probušila balon*) and English (*Sarah pierced the balloon*).

The pairs of pictures were black-and-white drawings, half of which were adopted from a normed database for psycholinguistic studies (Szekely et al., 2004) and the other half of which were drawn for the purpose of this study as the pairs of the normed pictures. One picture represented the correct choice depending on sentence meaning (e.g., the picture of a whole balloon for “*Sarah didn't pierce the balloon”* and “*Nobody pierced the balloon*” and the picture of a pierced balloon for “*Sarah pierced the balloon”*), while the other picture in the pair was the non-target competitor representing the incorrect choice. The size of each picture was 300 x 300 pixels. To mask the manipulation in the design, 20 filler sentences were mixed in with the target sentences, all presented in a fully randomized order. The filler stimuli also contained pairs of related pictures, but the co-presented linguistic input did not include any negation. The recordings for the fillers were compound sentences with coordinating conjunctions (e.g., *The patient wanted to bend his arm and the arm moved easily*). Each participant was given 84 trials in total, including 40 negative, 20 affirmative, 20 filler sentences and 4 training items.

### 5.3 Procedure

The experimental procedure was divided into two parts, based on the language of testing. The first round of experiments was conducted in Croatian, while the second round in English, with an inter-test gap of < 3 months for each participant. The experiments were programmed as web applications using the *jsPsych* (Version 6.3.1) and *Webgazer* (Papoutsaki et al., 2017) *JavaScript* libraries. We used the *jspsychread* package (Lukavský, 2023) for file processing. Testing took place in a quiet well-lit room, in the Laboratory for Psycholinguistic Research, University of Zagreb, using a desktop computer. The task was to listen to the sentences and look at the pictures appearing on the computer screen while the camera monitored participants' eye movements. Participants were instructed to remain as still as possible throughout the whole experiment. At the beginning of the test session, participants read the instructions displayed on the computer screen: “*You are going to see two pictures and hear a sentence. Pay careful attention to both. First, two pictures will appear side by side. Second, the pictures will disappear, and you will hear a sentence. After the end of the sentence, the pictures will reappear. Your task is to choose the picture that best corresponds to the sentence. Press the left arrow key if you choose the picture on the left, or the right arrow key if you choose the picture on the right. Decide as fast and as accurately as you can*”.

The experiment began with a 9-point calibration task to ensure accuracy of the webcam eye tracker. Then, a practice session followed to familiarize participants with the task. The sequence in a single trial consisted of a fixation cross in the center of the screen (1,500 ms), followed by a picture preview (2,500 ms), as shown in Figure 1. After preview, the pictures disappeared, and participants listened to the target or the filler sentences (2,500–3,000 ms). This is the “anticipation” time window during which participants saw a blank screen and received audio input. Once the audio recording was over, the pictures reappeared in their original positions and were displayed on the screen until the participant pressed a button. This is the “integration” time window during which participants saw the pictures in their original positions. The entire test session lasted ~40 min per participant. In sum, the Croatian L1 experimental design had two factors, *Condition* with three levels (positive, negative, nobody) and *Window* with three levels (preview, anticipation, integration). The stimuli consisted of 84 trials in total, including 40 negative, 20 affirmative, 20 filler sentences and 4 training items.

All data and codes used in the analyses are available at https://osf.io/9m5vd/.

### 5.4 Results: negation processing in L1 Croatian

**Figure 2:**
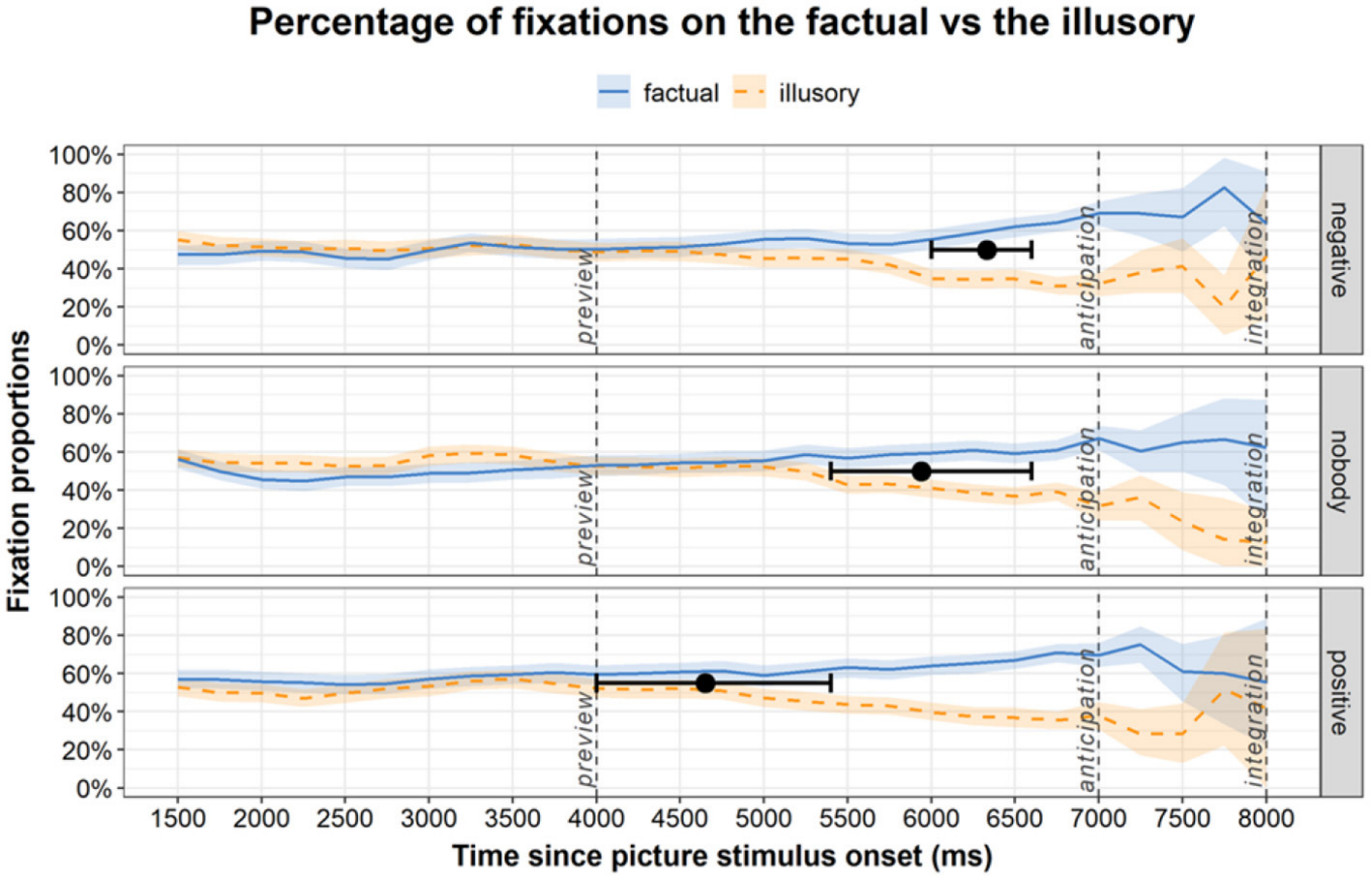
The blue and orange lines show the mean fixation proportions on the pictures showing the factual and the illusory state of affairs during preview, anticipation (audio in Croatian, no pictures), and integration (pictures in their original position). 95% confidence intervals are the shaded areas, the black points are the divergence points and the whiskers around them are their 95% percentile confidence intervals.

Figure 2 shows the proportions of fixations separately for three time windows, namely from the appearance of pictures until audio onset (preview window; 0–4,000 ms), from audio onset in the absence of pictures until the end of audio (anticipation window; 4,000–7,000 ms), and from picture reappearance at the end of audio (integration window; 7,000–8,000 ms). There were negligible differences in fixating on either picture in the preview window, suggesting that participants paid comparable attention to screening both the factual and the illusory pictures. The latter two windows provide insights into the time-course of negation processing. The anticipation window shows whether and when participants mentally simulate the linguistic information, indicated by directing looks to the position where they had seen the correct/factual picture earlier. In the integration window one can track the verification process during which participants double-check and revise the compatibility of linguistic input with the picture of their choice.

We first report descriptive statistics with the average proportions of fixations per condition and time window, complemented with mixed effects models to check whether fixations on the factual significantly differed from those on the illusory. For this purpose, a set of linear mixed models was built with *Condition* (negative, nobody, positive) and *Fixation target* (factual, illusory) as fixed effects, and with *Participant* and *Item* as random effects (lme4 package, R Studio, Version 4.1.1). The outcome variable was the total length of fixations, and the random effect structure was kept maximal. The default (treatment contrasts) was used. The formula was lmer(fixtotal ~ 1 + target ^*^ condition + (1 + target ^*^ condition | participant) + (1 + target | item). During anticipation, the average proportions of fixations to the correct picture (factual) were fairly similar in the two negation conditions; for negative concord (*nobody*), *M* = 57.2, *SD* = 45.9) and sentential negation (*negative*) (*M* = 55.3, *SD* = 46.1). In comparison, fixation proportions to the correct picture (factual) were greater in the control condition (*positive*) (*M* = 62.9, *SD* = 44.5) than in both negation conditions. Overall, proportions of fixations to the correct picture significantly exceeded fixation proportions to the incorrect picture (β = −0.19, *SE* = 0.05, *t* = −3.83, *p* < 0.001). During integration, the proportions of fixations to the correct picture in the control (positive) and sentential negation (negative) conditions were similar (control; *M* = 69.4; *SD* = 42.7; negative; *M* = 69.6; *SD* = 42.4), whereas the proportions of fixations was the lowest in the negative concord (nobody) condition (*M* = 65.2; *SD* = 44.5). Overall, fixation proportions to the factual were significantly higher than fixation proportions to the illusory (β = −0.37, *SE* = 0.05, *t* = −7.04, *p* < 0.001). The proportion of correct answers in the Croatian L1 dataset was high (100% for the *positive*, 99.69% for the *negative*, and 99.69% for the *nobody* condition).

The linear models above established that listeners looked preferentially at the target (factual), but we were also interested in *when* this preference first emerged and whether it emerged at different times between conditions. In the following step, we therefore used a divergence point analysis (Stone et al., 2021a,b) to determine whether the timecourse of fixations differed between conditions in Croatian L1, i.e., fixations on the factual vs. the illusory in each of the three conditions. The fixations were grouped into bins of 200 ms. Each bin was subjected to a linear model with weighted empirical logits (Barr, 2008; Veríssimo and Clahsen, 2014). We considered the onset of the experimental effect if there were significantly more fixations on the factual than on the illusory in the first of any three consecutive bins, consistent with a preference for the factual sustained for 600 ms. The data were then reshuffled within participants, conditions and time bins and the procedure was repeated 2000 times. Bootstrap confidence intervals were based on the distribution of the 2000 bootstrapped onsets and the percentile method. Bootstrapped divergence points and confidence intervals, superimposed on the fixation curves (Figure 2), were later in the negation conditions than in the *positive* condition (*M* = 4,651 ms, 95% CI 4,000–5,400 ms), with *nobody* condition (*M* = 5,940 ms, 95% CI 5,400–6,600 ms) being earlier than *negative* condition (*M* = 6,333 ms, 95% CI 6,000–6,600 ms). These are postverbal divergence onsets, the latter two suggesting that participants used the negation plus the verb to launch anticipatory fixations toward the target picture. Note that for the positive condition, the divergence point analysis suggested that there were two clusters of onset times, one shortly after the picture stimulus onset at 4,000 ms and one ~1,000 ms later. The mean and confidence interval of the onset estimate in Figure 2 take into account both of these clusters. Importantly, both clusters appear to be earlier than the onsets estimated for both the *negative* and *nobody* conditions.

**Figure 3:**
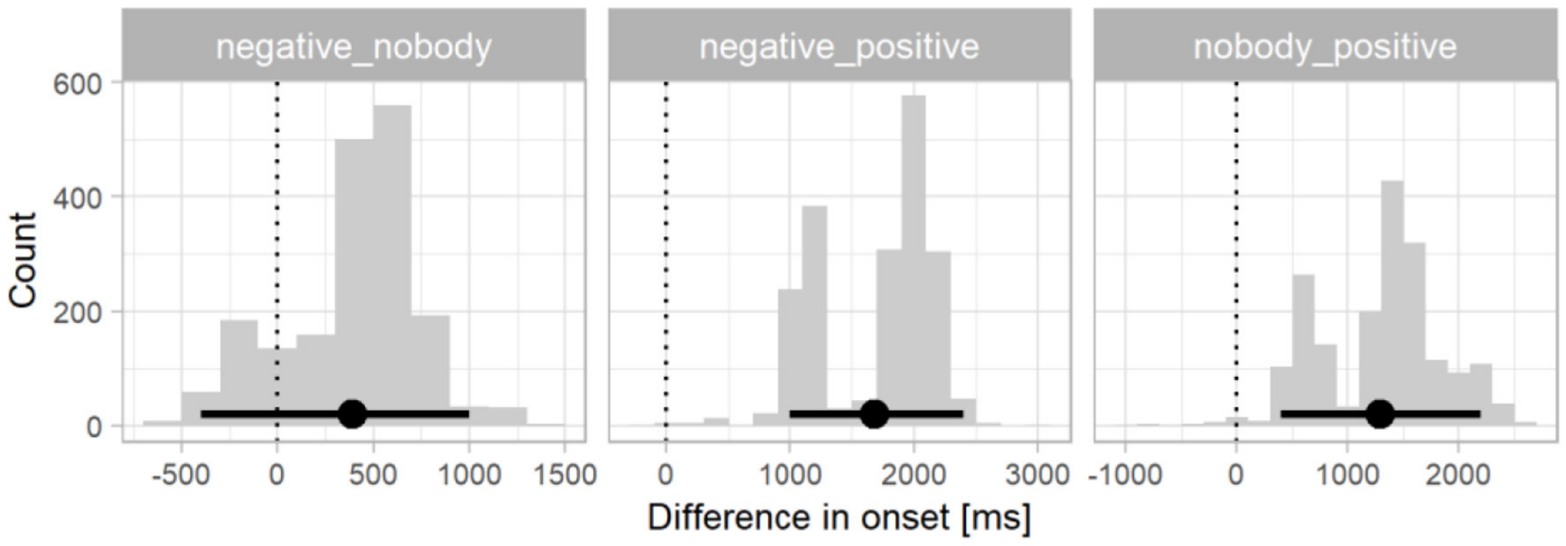
Differences in divergence point onsets for L1 Croatian. The x-axis shows the differences in the millisecond range in the onsets of divergence points, the y-axis shows the frequency of the differences in each time bin. The points and error bars indicate the bootstrap means and 95% confidence intervals. The dotted vertical lines indicate a zero difference between conditions.

Next, the differences in divergence points between the distributions in the three conditions were tested—*negative* vs. *nobody, negative* vs. *positive, nobody* vs. *positive*. The distribution of differences in divergence points between the three conditions is shown in Figure 3. The difference in divergence points between the *nobody* condition and *positive* condition (*M* = 1,290 ms, 95% CI 400–2,200 ms) was smaller than the difference between the *negative* and *positive* condition (*M* = 1,684 ms, 95% CI 1,000–2,400 ms). A smaller difference was observed between the *nobody* condition and the *negative* condition (*M* = 393 ms, 995% CI 400–1,000). As the 95% confidence interval for both comparisons with the *positive* condition did not contain zero, we can conclude that the onset of preferential looks toward the correct picture (factual) was significantly faster in the *positive* condition than in both negative conditions. The bimodality in these two difference distributions stems from the two clusters of onset times in the *positive* condition. Since the nature of the two clusters is unknown, we assume here that they belong to one process driving preferential looks to the target. The onset of the effect in the *nobody* condition and the *negative* condition did not differ significantly.

## 6 Experiment 2: processing negation in L2 English

### 6.1 Participants

A new group of 32 participants, also Croatian speakers of English with linguistic profiles closely matching those tested in Experiment 1, were recruited for Experiment 2 run in English.

### 6.2 Materials

Sets of English audio recordings and corresponding picture pairs were used in Experiment 2. The audios (3,000 ms) were English translations of the Croatian sentences from Experiment 1, read out by a native speaker of English. The English sentence types included sentential negation, negative quantifier negation, and affirmative controls. The picture pairs were identical across the two experiments and so were the numbers of trials (84 trials in total, including 40 negative, 20 affirmative, 20 filler sentences and 4 training items).

### 6.3 Procedure

The experimental procedures (Figure 1), as well as the procedures for analyzing the L2 English dataset followed the steps outlined for the L1 Croatian dataset. In sum, the L2 English experimental design also had two factors, *Condition* with three levels (positive, negative, nobody) and *Window* with three levels (preview, anticipation, integration). The stimuli were translations from Croatian, consisting of 60 critical items, 20 fillers, and 4 training items.

### 6.4 Results: negation processing in L2 English

**Figure 4:**
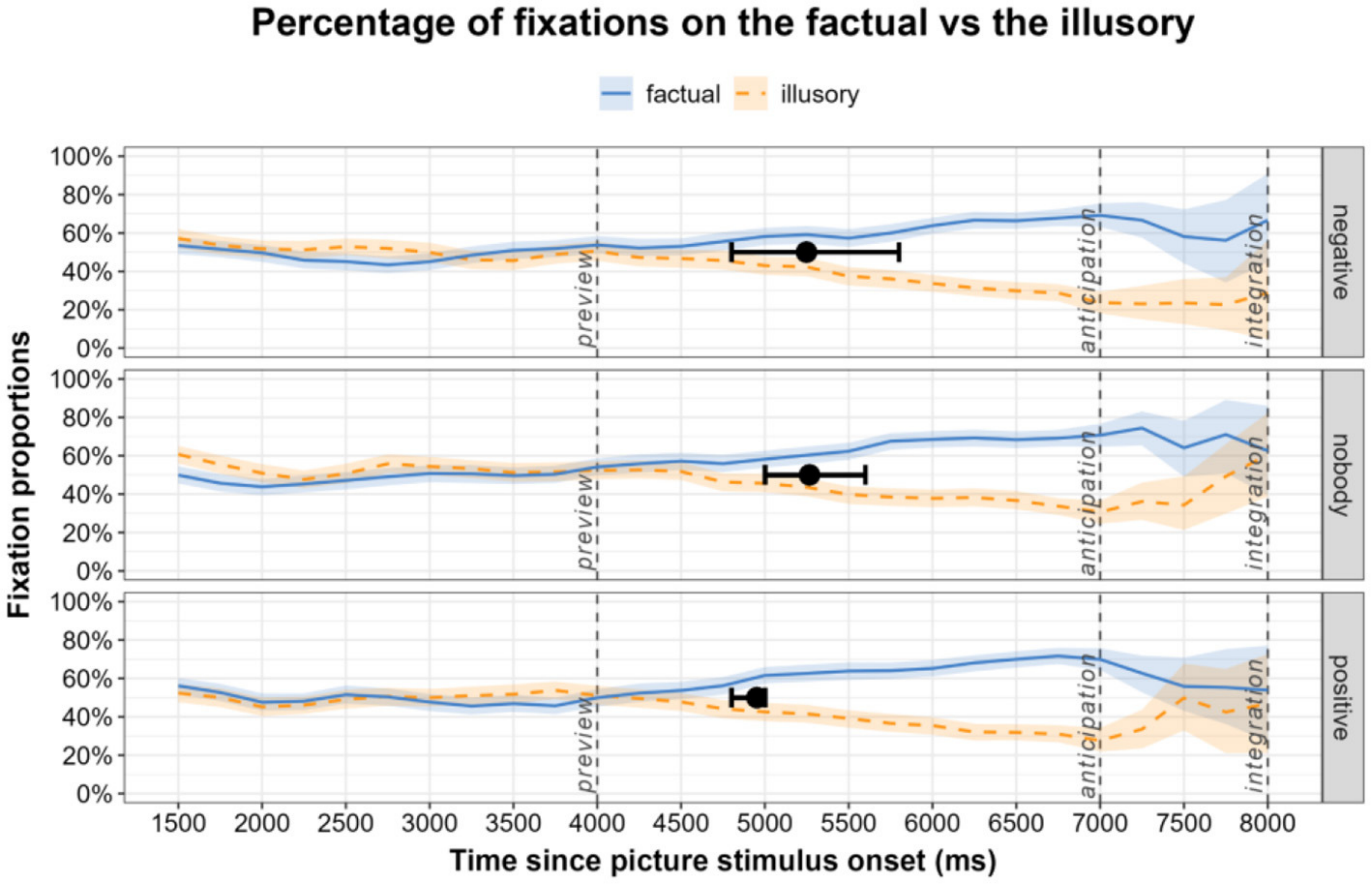
Mean fixation proportions on the pictures showing the factual and the illusory state of affairs during preview, anticipation (audio in English, no pictures), and integration (pictures in their original position). 95% confidence intervals are the shaded areas, the black points are the divergence points and the whiskers around them are their 95% percentile confidence intervals.

Descriptive statistics with the average proportions of fixations per condition and time window come first, followed by mixed effects models to test whether fixations between the factual and the illusory significantly differed. Figure 4 shows the proportions of fixations separately for preview (0–4,000 ms), anticipation (audio in English, 4,000–7,000 ms), and integration (7,000–8,000 ms). Fixations in the preview window show the screening of both pictures with comparable levels of attention. During anticipation in the absence of pictures, the average proportions of fixations to the factual picture were similar across all three conditions, namely for the control condition (*positive*) (*M* = 61.6, *SD* = 45.4), the sentential negation condition (*negative*) (*M* = 59.5, *SD* = 45.9), and the negative quantifier negation (*nobody*) condition (*M* = 62.2, *SD* = 45.4). Just like in the native language, proportions of fixations to the correct/factual picture in the L2 experiment significantly exceeded fixation proportions to the incorrect/illusory picture (β = −0.26, *SE* = 0.04, *t* = −6.39, *p* < 0.001). In the integration window, the average proportions of fixations to the correct picture (factual) were similar in the control condition (*positive*) (*M* = 65.7, *SD* = 44.0) and in the sentential negation (*negative*) condition (*M* = 66.6, *SD* = 44.2). The average proportions of fixation in the negative quantifier negation (*nobody*) condition were the highest (*M* = 70.8, *SD* = 42.0). Across conditions, fixations to the correct picture (factual) significantly exceeded fixations to the incorrect (illusory) (β = −0.48, *SE* = 0.05, *t* = −9.73, *p* < 0.001). The proportion of correct answers in the English L2 dataset was slightly lower than for the Croatian L1, but still very high overall (99.21% for the *positive*, 97.89% for the *negative*, and 98.29% for the *nobody* condition).

In the next step, we ran correlation tests to measure the strength of relationship between L2 proficiency scores and the degree of engagement in anticipatory fixations on the target picture. Three separate Pearson correlation coefficients were computed to assess the relations between L2 proficiency scores and the proportions of fixations on the factual in each condition. A significant positive correlation was found between the two variables in the *negative* condition, *r*_(30)_ = 0.44, *p* = 0.011. This was not the case in the *nobody* condition *r*_(30)_ = 0.17, *p* = 0.361 or in the *positive* condition *r*_(30)_ = 0.16, *p* = 0.375.

The following step was the analysis of how much the timecourses of fixations differed for the three conditions in L2 English. Analogous to the previous analyses of Croatian L1 data, fixations on the factual vs. the illusory underwent a divergence point analysis per condition. Bootstrapped divergence points and confidence intervals, superimposed on the fixation curves in Figure 4, were comparable across the conditions, with the fastest divergence points observed in the *positive* condition (*M* = 4,952 ms, 95% CI 4,800–5,000 ms) and similar divergence points in the *nobody* condition (*M* = 5,248 ms, 95% CI 5,000–5,600 ms) and the *negative* condition (*M* = 5,108 ms, 95% CI 4,800–5,800 ms). Both of the latter are postverbal divergence onsets, suggesting that in L2 English participants used the negation plus the verb to predict the target picture.

**Figure 5:**
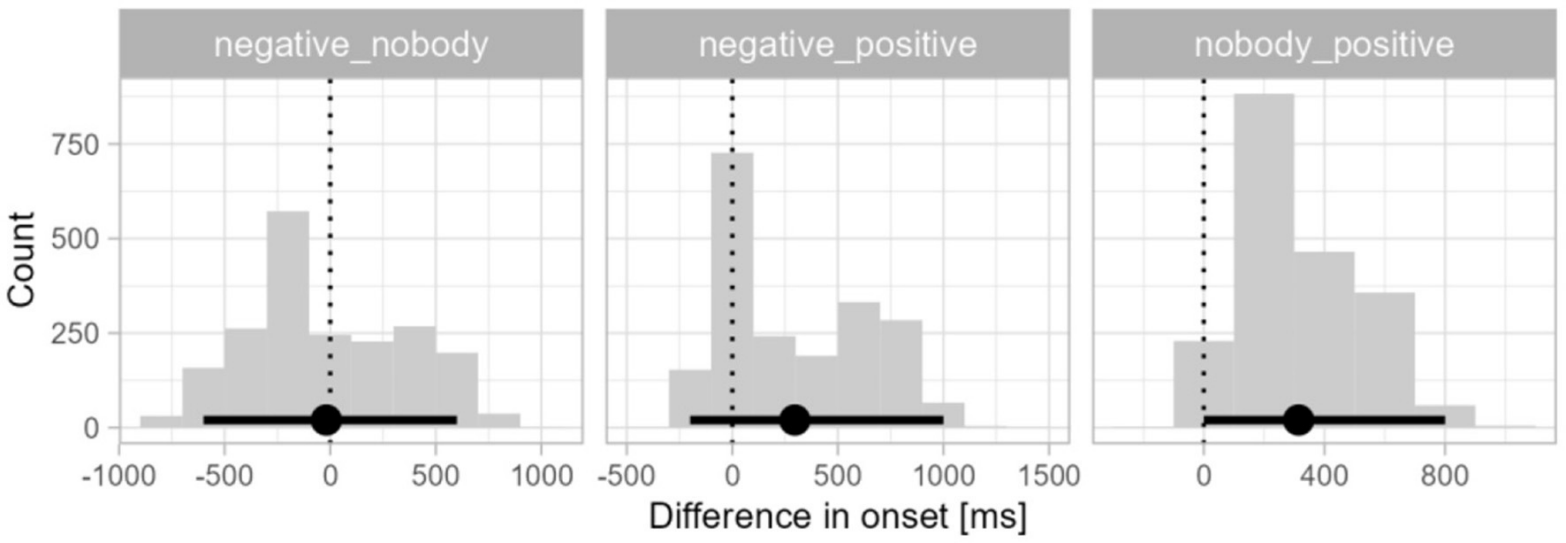
Differences in divergence point onsets for L2 English. The x-axis shows the differences in the millisecond range in the onsets of divergence points, the y-axis shows the frequency of the differences in each time bin. The points and error bars indicate the bootstrap means and 95% confidence intervals. The dotted vertical lines indicate a zero difference between conditions.

In the final step of the analyses we tested the differences in divergence points between the distributions in the three conditions in L2 English—*negative* vs. *nobody, negative* vs. *positive, nobody* vs. *positive*. The distribution of differences in divergence points between the three conditions is shown in Figure 5. The difference in divergence points between the two negation types was very small (*M* = 18 ms, 95% CI −600–600 ms), followed by the difference between the *negative* condition and the *positive* condition (*M* = 296 ms, 95% CI −200–1,000 ms). A slightly larger difference was observed between the *nobody* condition and the *positive* condition (*M* = 314 ms, 95% CI 0–800 ms). The 95% confidence interval for the first two comparisons contained zero, suggesting that the onset of preferential looks toward the factual picture in response to sentential negation (*negative*) and affirmation (*positive*) did not differ significantly. The 95% confidence interval for the nobody and positive condition difference bordered on zero and thus was not statistically significant, but the overall difference distribution did suggest that looks toward the factual picture in response to sentential affirmation (*positive*) may have been numerically faster than in response to negative quantifier negation (*nobody*).

### 6.5 Between-language differences in divergence points

**Figure 6:**
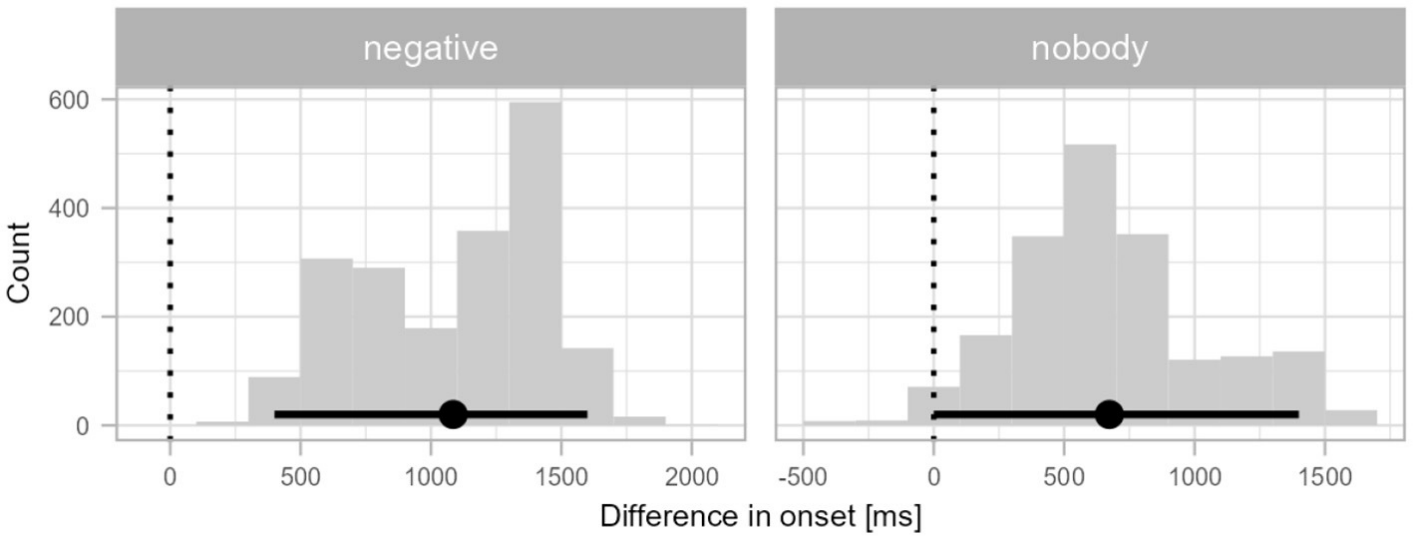
Differences in divergence points for the negation conditions between Croatian speakers' L2 (English) and L1. The x-axis shows the differences in the millisecond range in the onsets of divergence points, the y-axis shows the frequency of the differences in each time bin. The points and error bars indicate the bootstrap means and 95% confidence intervals. The dotted vertical lines indicate a zero difference between conditions.

While there were no significant differences across the three conditions in L2 English, the onsets of preferential looks in the negative conditions were qualitatively earlier than in L1 Croatian. To quantify this speed difference, we subtracted the onset distributions of each of the two negative conditions in the English data from those in the Croatian data. The difference distributions are presented in Figure 6. The onset of preferential looks to the factual picture was significantly slower in L1 Croatian by a mean of 1,085 ms in the *negative* condition (95% CI 400–1,600 ms). The 95% CI of the *nobody* condition bordered on zero and thus was not statistically significant although the distribution did suggest a numerical between-language difference of 673 ms (95% CI 0–1,400 ms).

## 7 General discussion

### 7.1 Main findings

The present visual world eye-tracking study using the blank screen paradigm examined negation processing in a first and a second language. We focused on the extent to which listeners anticipate the factual/negated states of affairs when they hear different types of negation unfold. Our rationale for L1 Croatian was to test whether generation of expectations about the factual will be more robust when negation provides more cues (negative concord) compared to just a single cue (sentential negation). Regarding L2 English, no such differences were predicted between the two types of negation, either due to structural differences (negative quantifier vs. sentential negation in English, i.e., single vs. single cue) or possibly as a result of a reduced ability to rely on anticipation processes in a second language. Three sets of findings emerged. First, listeners did mentally simulate the upcoming factual state of affairs across languages and conditions, which was documented in the anticipation window (blank screen) as a gradual increase in eye fixations on the factual but not on the illusory across conditions and languages. Second, unlike hypothesized, anticipation did not vary in L1 Croatian in accordance with the type of negation as the differences in the timecourses of launching anticipatory looks on the factual were not significant. And third, divergence points in the second language context occurred earlier than in the native language, which indicates an enhanced rather than reduced ability to generate expectations in a non-native language. We next discuss the theoretical contribution of these findings as well as their fit into the closest empirical context.

### 7.2 Contributions to theory

The main contribution of this study for second language research is new evidence that learners can predict the correct target when they process negation in a non-native language. This finding goes against the Reduced Ability to Generate Expectations (RAGE) hypothesis (Grüter et al., 2014), which was based on earlier L2 studies that failed to find support for predictive processing in an L2 context (e.g., Lew-Williams and Fernald, 2010; Martin et al., 2013). Through a test of prediction abilities in Croatian learners of English, we show that anticipation effects in L2 processing can emerge not just when the L1 and L2 are structurally similar as previous work might suggest (Dussias et al., 2013; Foucart et al., 2014; Van Bergen and Flecken, 2017). Although translation equivalents, negative concord in Croatian structurally differs from negative quantifier negation in English to an extent that some might say L1-based anticipation processes in this case would be useless for L2 online sentence comprehension. Structural cue overlap between languages turns out to be facilitatory in much of previous work, but it is not a necessary condition for anticipation effects in L2 to occur (Kaan and Grüter, 2021). There is growing evidence that the transfer of using L1 cues predictively in an L2 is not limited to straightforward structural overlaps (Foucart, 2021; Hopp, 2016). The eye fixation results in the second language context suggest that a partial overlap in the negative quantifier *nobody* could serve as a sufficiently reliable cue to predictively launch looks toward the factual picture. Whether the same predictive machinery operates bidirectionally or not remains to be investigated. A design with English learners of Croatian would be informative to tell in future research.

Why did fixations diverge earlier in L2 than in the L1, contrary to our hypothesis? These findings have direct implications for the Unified Competition Model (MacWhinney, 2005, 2008, 2012). We advocate that on top of negation structures, an additional process, *multiple hypothesis upkeep*, factored into cue weighting in L2 and L1 and resulted in the observed L2-L1 variations in cost-benefit trade-offs. In the stronger L1, arguably more cognitive resources than in the weaker L2 could be allocated to upholding multiple hypotheses for a longer time about which picture may be the correct one. Sustained activation of alternative hypotheses (e.g., of a clean plate vs. a dirty plate) would work particularly well for processing numerous filler sentences with unrealized events that were used for masking purposes (e.g., *The waiter tried to clean the plate, but the plate remained dirty*). Sentences with ambiguous referents until quite late into the sentence could have discouraged early anticipation in the L1, and, consequently, delayed the utility of prediction throughout the experiment (divergence points in the *negative* and *nobody* conditions came at ~2,000 ms during sentential input, a timepoint at which a denial of event realization would be a likely sentence continuation). In the L2, however, cognitive resources needed for linguistic decoding could have driven early commitments to the most plausible prediction based on the first available cue. Placing different weights on cues in an L2 as in an L1 may be driven by high uncertainty due to fast processing speed needed for listening, or to less specified linguistic representations (Kaan and Grüter, 2021). In the context of negation processing, it is likely that L2 listeners have relied on cues from their prior language experience, that is, they have utilized prototypical associations between *nobody/didn't* as indicators of negated states of affairs, mapped these associations from their L1 to their L2, and committed to them early to free up resources useful to decode the rest of the fast-unfolding input stream. The finding that differences between negation conditions were greater in L1 Croatian than in L2 English confirms our hypothesis about the non-parallel nature of processing negation changing as a function of structural cueing. We observed that the timecourses of negation processing varied more in L1 Croatian than in L2 English, but not significantly. We hypothesized a difference based on greater cue variation in Croatian (negative concord vs. sentential negation, i.e., double vs. single cue respectively) than in English (negative quantifier vs. sentential negation, i.e., single vs. single cue), but the results did not confirm this prediction.

One possible explanation for the absence of a processing difference between negative concord and sentential negation in Croatian is that the two negative cues may have been processed as a single unit. This account invites comparisons with other examples where negative concord lost, or is losing, one negative item. For instance, two negative cues in negative concord were used to express a single semantic negation in Old English as well as in Middle English (Noland, 1991), and later one negative item got dropped in the diachronic process of simplification. A similar process can be traced in modern French, where *ne* is often omitted [e.g. *Je (ne) suis pas allé*e “I didn't go”] depending on sociolinguistic factors (Dewaele, 2004). This historical perspective invites future experimental research to examine, for instance through a self-paced version of the gradient acceptability judgement task (Blanchette, 2017), whether Croatian native speakers process the two negative items in Croatian negative concord as a single cue. Additionally, in related research, one remaining question is what makes a certain linguistic cue salient. Is it its position in a sentence, the number of lexical items that express negation, or something else? For example, could a Croatian sentence *Nikome ništa ne dugujem*, “I owe nothing to anyone”, be considered more salient and hence processed faster than a “regular” negative concord sentence, solely because it contains three negative items? The notion of cue saliency in negation processing remains an intriguing question, both from a theoretical and a methodological point of view.

As for the between-language differences, could an explanation for earlier divergence points in L2 English than L1 Croatian be that negation in English is simply easier to process than negation in Croatian? Comparisons of divergence points in L2 English in the present study and L1 English in Vanek et al. (2024) may be informative in this respect. Using the same paradigm and the same materials across studies, fixations diverged around the same points in L2 English as in L1 English in the *nobody* condition: L2 DP = 5,248 ms, 95% CI 5,000–5,600 ms, vs. L1 DP = 5,224 ms, 95% CI 4,800–5,800 ms. Such close proximity of DPs across L1 and L2 may suggest that negation using the negative quantifier *nobody* is easier to process in English than it is in Croatian. However, in the *negative* condition, fixations diverged earlier in L2 DP = 5,108 ms, 95% CI 4,800–5,800 ms than in L1 DP = 5,236 ms, 95% CI 5,000–5,400 ms, which could be interpreted as some support for the idea of earlier commitments in L2 to free up resources for subsequent input decoding. Caution is due though as the wide confidence intervals warn against firm conclusions in this respect. A clearer picture emerges when comparing DP differences between the two negation conditions. While they were only 6 ms apart in L2 English (M = 18 ms, 95% CI −600 to 600 ms) and L1 English (M = 12 ms, 95% CI −600 to 400 ms), the difference was as much as 393 ms (CI 400–1,000) in L1 Croatian, aligning with the view of language-specificity in processing negation can vary as a function of structural cueing.

Increases in overall L2 proficiency often emerge as indicators of the degree to which L2 speakers engage in predictive processing (e.g., Chambers and Cooke, 2009; Dussias et al., 2013). One of our findings aligns with this idea. Namely, for sentential negation processing in L2 English, we observed that higher proficiency in L2 correlated with increases in the proportion of anticipatory eye-fixations. The structural features of sentential negation largely overlap between the L1 and the L2, which seems to provide a reliable cue that more proficient L2 speakers could utilize for generating more predictions. However, the predictive use of a less reliable cue did not mirror this pattern. This can be seen in the case of negation with a negative quantifier in L2, where L2 proficiency and prediction generation did not correlate. We interpret this absence of a correlation as a result of little overlap between the structural cues in L1 and L2. Our findings from negation with a negative quantifier in English add to a considerable portion of recent research which points in the direction that the relationship between L2 proficiency and prediction ability is not automatic (e.g., Dijkgraaf et al., 2019; Kim and Grüter, 2021; Domazetoska and Zhao, 2024). Nevertheless, caution is needed in interpreting the presence or absence of an L2 proficiency effect on prediction ability. To substantiate this need for caution, no correlation between proficiency and prediction emerged in the positive condition, which was arguably the easiest one to process. A possible reason could be that many affirmative sentences in the experiments were fillers with defeated realizations in the second clause. Therefore, in the course of the task, such fillers could reduce cue reliability of verbs with a positive polarity. Comparisons across conditions to see when anticipatory looks were more likely show that L2 proficiency matters for processing efficiency, but only when the cues are reliable.

Divergence point analyses revealed cumulative increases in looks on the factual across conditions and in L1 as well as L2. The timecourse data shows no point, neither during anticipatory nor during integratory processing, at which fixations on the illusory would exceed those on the factual. This finding poses problems for the two-step model of negation processing (Coso and Bogunović, 2016, 2019; Kaup et al., 2006, 2007). Instead, we interpret the results, particularly from the anticipation time window, as support for the idea that negation can be understood through mental simulation of the factual/negated state of affairs directly, without a necessary detour via the illusory/positive (Orenes et al., 2014; Tian et al., 2010). Direct processing of negation both in Croatian as a first and English as a second language may be viewed as an indicator of a successful optimization of processing efficiency through a combination of available resources, and first as well as second language knowledge. One further point to add is that the bootstrapped divergence points and the resulting between-condition difference distributions were not normally distributed, meaning that the onset differences between conditions should be interpreted with caution. Nonetheless, the study theoretically contributes on various levels, providing new insights for crosslinguistic cue-based theoretical accounts and also for models of negation processing. All in all, the findings conflict with the predictions of the RAGE hypothesis, they point to limited variation in cue strengths that enrich a Unified Competition Model for negation processing, and they also highlight that one can comprehend negation in a single step in the first as well as in the second language.

### 7.3 Limitations and avenues for future research

It was reassuring to observe that no significant differences in fixation patterns emerged across conditions and languages throughout the entire picture preview stage (2,500 ms). This is an important design validity check aiding the theoretically motivated claim that the differences in fixations are attributable to cue strength linked to negation types rather than other factors such as learning effects. Should learning of probable item types have influenced performance, one could expect that the overall higher frequency of negative compared to affirmative sentential input would have acted as iterative feedback loops influencing the downward flow of predictive processing already during the preview stage.

Perhaps the clearest set of findings from the study is that, across negation types and languages, participants tended to direct their attention toward the picture with the factual state of affairs instead of focussing on the illusory state of affairs first. One might wonder to what extent the chosen paradigm activated mental representations of the factual even before anticipatory eye movements became relevant. The experimental design affords ample preview time during which participants are exposed to two images illustrating two contrasting states in which a target object could be, like a balloon being either pierced or intact. Two related points emerge. First, given that the sentences consistently describe an action that results in one of the states, participants have a good chance of predicting the verb just by looking at the pictures. It is true that this may be the case for a number of trials with high predictability of the type of action, like *chopped the onion, tore the exam, sliced the lemon*, or *dropped ice cream*. Even though predictability of the events being realized or not realized was low up until the verbal input, predictability of the type of action from pictures was not controlled. It is a limitation that future studies could address, e.g., by asking participants to guess the most likely verb linked to each picture pair. Then, the ratio of correct guesses could be used as a fixed factor in statistical models to wash out possible type-related noise. Second, there is another portion of stimuli for which the type of action may be less guessable from just looking at the pictures, like *open/close envelope, iron/wrinkle sweater, light/extinguish fire, shave/grow a beard*. Still, some might wonder whether even for such events the results could reflect exercise effects rather than manipulation of linguistic cues because exposure to the same pictures presented with the same verbs was as many as six times in the course of the experiments (three times per language). Some training effects cannot be ruled out. Had they been robust though, they would have triggered a divergence of fixation curves already at the later stage of the picture preview, which was not observed.

Why did participants move their eyes so quickly somewhere when there was nothing to see? Initially, the visual scene was shown, but it was subsequently removed before the target sentence was presented. Prevalent fixations launched toward the earlier shown location of the factual state suggest that eye movements depend on mental representations of the factual state regardless of its actual presence. This can be explained if we assume an internally maintained visual scene with spatial coordinates stored in episodic memory for each item (Richardson and Spivey, 2000). Activating this memory with language automatically triggers the component that encodes the item's location, which in turn guides the eyes to that specific location. The timecourse of eye fixations when nothing was on the screen is most informative at points where fixations diverge. The fixations typically diverged at points during/after the event-denoting verb (*pierced/didn't pierce, broke/didn't break*). To probe further into the mapping between language and the visual world, future studies might benefit from aligning the presentation of the key information more strictly (e.g., playing the critical verb not later in the negative condition, only after *didn't*, but exactly at the same time across conditions). Complementary to the memory-based spatial coordinates idea, another plausible account of why eye movements got launched onto a blank screen comes from predictive processing (Lupyan and Clark, 2015). Participants had to choose the picture that matches the sentence meaning, and the picture only reappeared after the sentence had finished. To rapidly deal with unfolding linguistic input and match it with the previously presented visual data, participants seem to have integrated their memory-based spatial coordinates to build “top-down” predictions so that these could facilitate selection of the matching picture after its reappearance. The timecourse of fixations on the blank screen supports the idea that eye movements reflect a predictive process. Divergences usually occurred during or past the verb (*pierced/didn't pierce*) rather than just after hearing the depicted referent (*balloon*), so before all the information to choose the correct picture was available. Fixations on the factual before the referring expression was mentioned suggest that participants connected their memory traces about object locations with verb phrase semantics to build predictions about the matching state of the object. It could be advantageous to have a predictive processing more clearly separated from integration, for instance by shortening the prediction window to end just before the referring expression gets mentioned. A further improvement for future work may be to also include non-contrasting states of different objects (*coconut, newspaper*) combined with a verb (e.g., *eat*) where only one object satisfies the selection restrictions.

Participants reported exposure to English through social media, where non-standard uses of negation abound. It might be informative in future work to pre-screen participants' acceptability and comprehension of negative concord in English. Additional data of this type would be helpful to check if acceptability and comprehension of a negation variant different from what is learnt in school changes as a function of contextual priming in L2, as it does in L1 (Blanchette and Lukyanenko, 2019). This data type could also help shed light on whether the degree of social media use can predict acceptability of non-standard negation.

## 8 Conclusion

In sum, we advocate that the factual/negated state of affairs during sentence comprehension can be processed directly in both the first and the second language. Our evidence comes from anticipatory looks launched toward the upcoming target, in cases when negation types exhibit crosslinguistic overlap (sentential negation) as well as when the L1 and L2 structurally differ (negative concord in Croatian vs. negative quantifier in English). Cumulative increases of fixations on a blank screen (Altmann, 2004) in anticipation of the factual, but not the illusory, suggest direct mental simulation of the negated state of affairs. No significant variation in fixation onsets changing with negation type in L1 Croatian show that anticipation effects are not stronger when there are more negative items to rely on. Lack of variation in fixation onsets across affirmation and negation types in L2 English point to other than L1-driven processing routines. These are interpreted as adaptations to optimize processing efficiency in a second language context.

## Data Availability

The datasets presented in this study can be found in online repositories. The names of the repository/repositories and accession number(s) can be found below: https://osf.io/9m5vd/.
